# Fibronectin and Its Role in Human Infective Diseases

**DOI:** 10.3390/cells8121516

**Published:** 2019-11-26

**Authors:** Pietro Speziale, Carla Renata Arciola, Giampiero Pietrocola

**Affiliations:** 1 Department of Molecular Medicine, Section of Biochemistry, University of Pavia, Viale Taramelli 3/b, 27100 Pavia, Italy; giampiero.pietrocola@unipv.it; 2 Laboratorio di Patologia delle Infezioni Associate all’Impianto, IRCCS Istituto Ortopedico Rizzoli, via di Barbiano 1/10, 40136 Bologna, Italy; carlarenata.arciola@ior.it; 3 Department of Experimental, Diagnostic and Specialty Medicine, University of Bologna, via San Giacomo 14, 40126 Bologna, Italy

**Keywords:** fibronectin, extracellular matrix, bacteria, adhesin, virulence factor, vaccine

## Abstract

Fibronectin is a multidomain glycoprotein ubiquitously detected in extracellular fluids and matrices of a variety of animal and human tissues where it functions as a key link between matrices and cells. Fibronectin has also emerged as the target for a large number of microorganisms, particularly bacteria. There are clear indications that the binding of microorganism’ receptors to fibronectin promotes attachment to and infection of host cells. Each bacterium may use different receptors which recognize specific fibronectin domains, mostly the *N*-terminal domain and the central cell-binding domain. In many cases, fibronectin receptors have actions over and above that of simple adhesion: In fact, adhesion is often the prerequisite for invasion and internalization of microorganisms in the cells of colonized tissues. This review updates the current understanding of fibronectin receptors of several microorganisms with emphasis on their biochemical and structural properties and the role they can play in the onset and progression of host infection diseases. Furthermore, we describe the antigenic profile and discuss the possibility of designing adhesion inhibitors based on the structure of the fibronectin-binding site in the receptor or the receptor-binding site in fibronectin.

## 1. Introduction

The extracellular matrix (ECM) of animal and human tissues is a complex meshwork of secreted proteins immobilized in the space between cells. The global composition of the extracellular matrix proteins constitutes the “matrisome” [1]. The matrisome essentially consists of the core “matrisome” (around 300 proteins), which includes macromolecules such as collagens [2], proteoglycans [3] and glycoproteins, and “matrisome-associated proteins” that comprise a number of functionally and structurally different proteins ranging from annexins and complement proteins to enzymes and growth factors [4]. Collagens provide structural strength to ECM and contribute to the mechanical properties, organization, and shape of tissues. Proteoglycans are interspersed among the collagen fibrils in different ECMs and confer space-filling and lubrication functions. Glycoproteins perform a variety of functions including ECM assembly, cell adhesion to ECM, and signaling to the cells. The best studied glycoproteins of ECM are laminins [5,6], fibronectins [7] thrombospondins [8], fibrinogen and vitronectin [9]. Many ECM components play a vital role in guiding the migration and polarity of cells, cell proliferation, differentiation, and stem cell state. ECM components also function as a link between cells and their ECM through membrane-spanning receptor proteins called integrins. Integrins are heterodimers made up of one α chain and one β chain both of which are type I membrane proteins. In vertebrates there are 18 α and 8 β subunits that can assemble noncovalently into 24 different receptors with different specificity for ligands and distribution in tissues. The *N*-terminal halves of α and β subunits associate to form the binding region for ECM components, while the remaining portions form two rod-shaped tails that span the plasma membrane [10,11].

As a ubiquitous constituent of animal tissues, the ECM can also serve as a substrate for the adherence of colonizing bacteria. To this end, bacteria express a variety of surface proteins, many of which are adhesins belonging to the family of MSCRAMMs (microbial surface components recognizing adhesive matrix molecule). The term MSCRAMM refers to cell wall-anchored proteins that “have structural and functional similarities and a common mechanism of ligand binding performed by two adjacent IgG-like folded domains” [12]. The ECM proteins that function as MSCRAMM ligand include collagen, fibronectin (Fn), fibrinogen, and laminin. The best characterized bacterial fibrinogen-binding proteins include ClfA and ClfB (clumping factor A and B), the collagen/laminin-binding protein CNA and the Fn-binding proteins FnBPA and FnBPB from *S. aureus* [12,13]. MSCRAMMs usually have a multidomain organization and each domain can bind to a diverse array of host ligands. For example, the A domain of FnBPB (Fn-binding protein B) besides fibrinogen [14] recognizes and binds to elastin [15], plasminogen [16], and histones [16], while the C-terminal repetitive region interacts with Fn [17].

The present review aims to provide key information on the variety of bacterial Fn-binding proteins and the role they can play in the colonization of host tissues and the onset of infectious diseases. We also provide evidence of the potential use of these adhesins as therapeutic tools against pathogens.

## 2. Functional and Structural Properties of Fn

**Figure 1:**
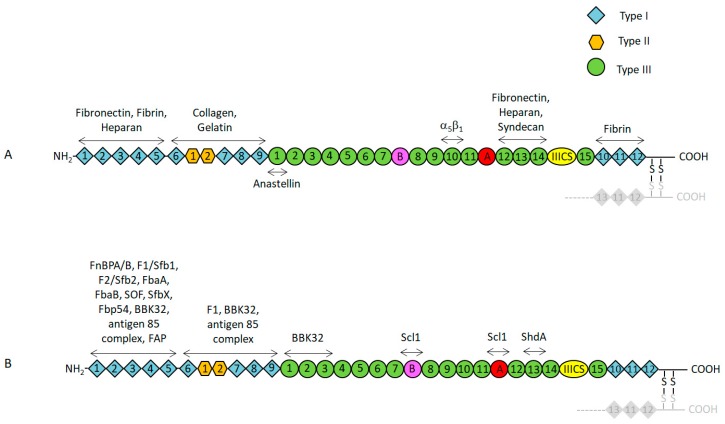
(**A**) Schematic representation of plasma/cellular fibronectin. Type I, II, and III modules are denoted by squares, hexagons, and circles, respectively. The IIICS module is represented by an oval. Alternative spliced extra domains A, B, and IIICS are shown in pink, red, and yellow, respectively. Binding sites for extracellular matrix proteins and the integrin α5β1 binding site are reported. The localization of anastellin activity in the FIII_1_ is also indicated. (**B**) Cartoon representation of fibronectin as reported in A with the indication of the binding regions for several bacterial receptors.

Fn is a multidomain glycoprotein (440 kDa) found in almost all tissues and organs of vertebrates. Fn plays an important role in several biological processes such as adhesion to ECM, differentiation, growth, and migration of cells. Furthermore, Fn is involved in embryonic development, blood clotting, and wound healing [18]. Alteration of Fn expression, degradation, and organization have been related to several pathologies, including oncogenic transformations and fibrosis [18]. The protein is generated by a single gene and alternative splicing of a single pre-mRNA leads to the production of at least 20 isoforms in humans. Fn exists in soluble form in various body fluids and as an insoluble component of many extracellular matrices and basement membrane. In soluble form, intramolecular interactions result in a more compact conformation of Fn, while the protein becomes extended when deposited in extracellular matrix in a process known as Fn assembly. Soluble Fn is produced by hepatocytes and secreted into the bloodstream, whereas fibroblasts and endothelial cells synthesize insoluble cellular Fn. Fn is a protein dimer, consisting of two nearly identical monomers linked by a pair of disulfide bonds. Each subunit carries 5–7 asparagine-linked carbohydrate side chains and one or two O-linked chains. It has been proposed that glycosylation protects Fn from proteolysis [18]. Fn has a modular architecture composed of a combination of three different types of homologous domain, i.e., type I (FnI), II (FnII), and III (FnIII). Specifically, each monomer consists of 12 type I repeats (40 aa residues each), 2 type II repeats (60 aa each), and 15–17 type III repeats (90 aa residues each) [18,19,20]. The repeating modules of Fn monomers fold independently with 25%–30% β-structure and no α-helix. The structure of cellular Fn can include two variable proportions of alternatively spliced FnIII modules (EIIIB and EIIIA, also termed EDB and EDA, respectively) and one FnIII connecting segment (IIICS), while the soluble form lacks these alternatively spliced type III repeats. The EDA and EDB segments are not expressed in healthy adult tissues but are detectable in wound beds and solid tumors [21,22]. Extended polypeptide segments in certain parts of each chain are highly susceptible to proteolysis, which generate a series of protease-resistant domains, each comprising several of the repeating modules and each containing several binding sites for different specific ligands. Five independently folded type I modules (modules FI_1–5_) constitute the *N*-terminal domain (NTD) which binds heparin, fibrin, and bacterial receptors (Figure 1A,B). This domain also plays an essential role in Fn matrix assembly by binding two major sites within Fn: One site in native III_2_ and a second cryptic site that is exposed in denatured III_1_. Matrix assembly also requires integrins and molecules that connect integrin to the cytoskeleton. The region immediately downstream of the FI_1–5_ domain, consists of the combination of FnI_6_FnII_1–2_FnI_7–9_ repeats and binds gelatin/collagen. The wide region including 15 constitutive type III repeats comprises a set of modules for binding other extracellular molecules (e.g., heparin), the RGD (FnIII_10_), and synergy (FnIII_9_) sequences for integrin binding and modules required for fibrillogenesis. Anastellin is a small recombinant carboxy-terminal fragment of the first FnIII module (FnIII_1_) from human Fn. Anastellin displays anti-tumor, anti-metastatic, and anti-angiogenic properties in vivo and is capable of binding and converting the soluble Fn into insoluble fibrils (superfibronectin) that structurally and functionally resemble Fn deposited in the extracellular matrix by cells [23,24]. The extreme C-terminal of Fn (FnI_10–12_) contains a second fibrin binding site and a cysteine residue necessary for the interchain disulphide bond. Although the NTD is the main bacterial binding site, additional sites with binding activity for specific bacterial species are also present in Fn (see below) (Figure 1A). 

## 3. Properties of Bacterial Fn-Binding Proteins

### 3.1. Fn-Binding Proteins from Staphylococcus aureus as a Prototype of a Wide Class of Bacterial Adhesins

*Staphylococcus aureus* is one of the most important human pathogens, causing a variety of diseases, including skin and soft tissue infections, osteomyelitis, endocarditis, surgical site infections, pneumonia, and sepsis [25,26].

**Figure 2:**
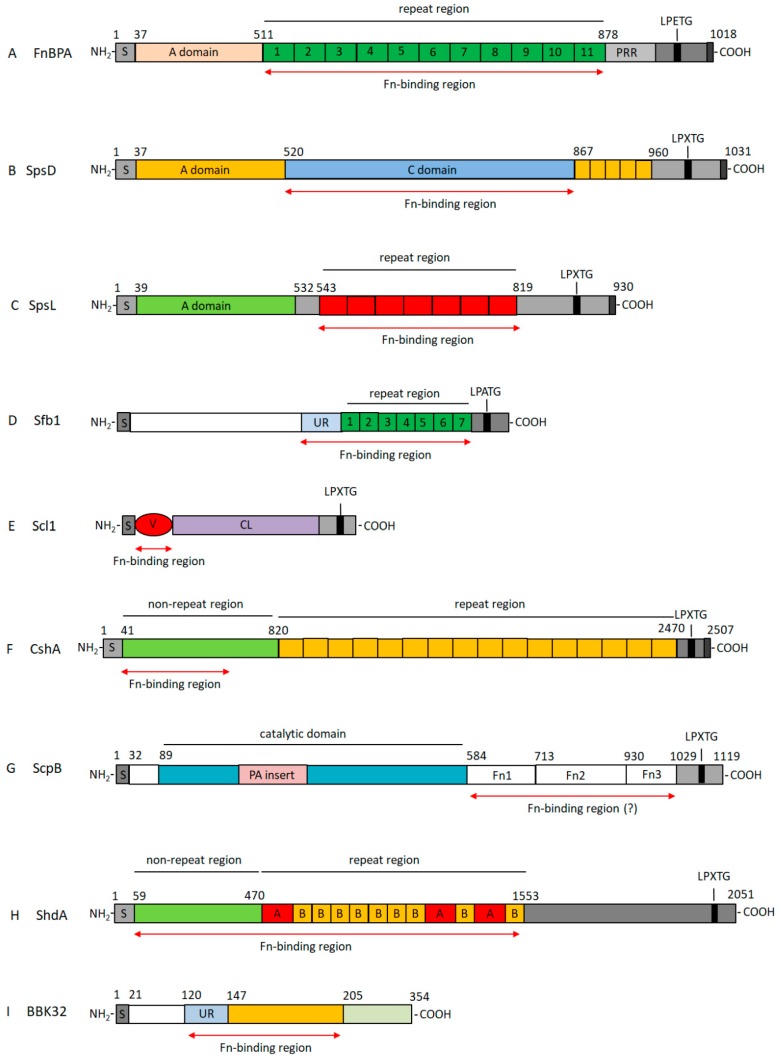
Representation of the structure of surface proteins from Gram-positive and Gram-negative bacteria. The reported proteins are cell wall-anchored proteins that contain a signal sequence (S) at the N-terminus and a sorting signal at the C-terminal end. BBK32 is not an LPXTG protein and is anchored to the bacterial surface by an *N*-terminal lipobox. Each protein is identified with a capital letter (**A**–**I**). Domains involved in fibronectin binding are also shown. For further details, see the text. The drawings shown here represent those for which the organization and fibronectin binding sites have been defined.

Most strains of *Staphylococcus aureus* express two related Fn-binding proteins FnBPA and FnBPB (Fn-Binding Protein A and B), which are encoded by closely linked genes [17]. The two proteins contain *N*-terminal signal sequences that promote protein localization at the cell surface and a sorting signal, which comprises an LPXTG (Leu-Pro-X-Thr-Gly where X stands for any amino acid) motif that is crucial for cell wall anchoring, a transmembrane hydrophobic domain and a stretch of cytosolic positively charged residues, at the C-terminus (Figure 2A). FnBPA and FnBPB possess fibrinogen-and elastin-binding capacities mediated by the *N*-terminal A domain region through a variation of the DLL (Dock, Lock and Latch) mechanism [27]. The DLL mechanism is based on the observation that MSCRAMM family proteins have an *N-*terminal A region composed of three distinct folded subdomains N1, N2, and N3. The N2 and N3 subdomains concur to create a hydrophobic trench that accommodates peptide ligands, in the case of ClfA, FnBPA, and FnBPB the extreme C-terminus of the γ chain of fibrinogen. Then, a conformational change is triggered, resulting in the redirection of the short unstructured C-terminal extension of the N3 subdomain, folding of this extension over the bound peptide and locking in place. In the final latching step, the C-terminus of the extension interacts with the N2 subdomain and forms a β-strand complementing a β-sheet in the N2 subdomain.

Linking the A region to the wall spanning region is a long, intrinsically disordered Fn-binding repeat (FnBR) domain (11 repeats in FnBPA and 10 in FnBPB) (Figure 2A). The Fn-binding repeats bind to the *N*-terminal domain (NTD) of Fn (Figure 1B). When bound, the FnBP acquires an ordered secondary structure by adding an antiparallel strand to the three-stranded β-sheets of the four sequential Fn type I modules. The tandem array of β-strands is called the β-zipper mechanism [28,29]. Of the 11 Fn-binding repeats six bind to Fn type I modules with high affinity and stoichiometry of FnBP binding to Fn is estimated to be between six to nine Fn molecules per FnBP [30,31]. 

A study performed by Casillas-Ituarte et al. identified nonsynonymous, single nucleotide polymorphism in the *fnbA* gene. Specifically, the aa substitutions in FnBR-5 and FnBR-9 of FnBPA from isolates of patients with infected cardiac devices and infective endocarditis confer an increased binding affinity for Fn in *S. aureus* suggesting that strains with these substitutions have an enhanced capability to evade host defenses and/or colonize damaged tissue or implants. Notably, amino acid substitutions in the FnBR region of FnBPA alter the strength of the fibrinogen interaction with the A domain of FnBPA possibly in a way that impacts on the DLL mechanism [32]. 

*S. aureus* and a number of other pathogens can invade non-professional host phagocytic cells via a mechanism whereby adhesin-bound Fn bridges interact with α_5_β_1_ integrin [33,34]. Along this line it has been found that upon binding to Fn, FnBPA disrupts specific intermolecular contacts in the *N*-terminal domain of Fn resulting in global structural organization, exposure of the cryptic RGD binding site in the 10th FIII module, and consequent promotion of interaction of Fn with α_5_β_1_ [35]. The formation of the three-component FnBPA-Fn-α_5_β_1_ integrin complex is dramatically potentiated when *S. aureus* cellular invasion is performed under shear forces [36].

These events trigger “outside in” signaling process leading to integrin clustering [30,37] and the formation of focal complexes on the host cell surface followed by eventual internalization of Fn-coated *S. aureus* cells via a zipper-like mechanism [38,39].

*S. aureus* expresses other Fn-binding proteins that further concur to strengthen adherence of the bacterium to the host tissues. The largest of these is 1.1 MDa Ebh (for ECM binding protein homologue), a surface protein consisting of several distinct regions including a signal peptide, an *N*-terminal domain annotated as a hyperosmolarity resistance domain, seven repeats of the 54-residue FIVAR domain (possibly associated with polysaccharide binding), 50 repeats of the 123 residue FIVAR-GA domain involved in albumin binding, seven repeats of DUF1542 (a 72-residue domain of unknown function), a putative SMC (structural maintenance of chromosomes) domain, a transmembrane domain, and, lastly, a positively charged cytoplasmic stretch [40]. Recent studies suggest that Ebh plays a role in cell growth and envelope assembly [40] and contributes to structural homeostasis of the bacterium forming a bridge between the cell wall and cytoplasmic membrane [41]. The central region of Ebh was found to specifically bind to Fn [42]. 

Another Fn-binding protein from *S. aureus* is protein Eap (Extracellular adherence protein) released in the growth environment. Eap, termed also MAP (Major histocompatibility class II Analogous Protein), is a 50–70 kDa protein that consists of multiple (most often four or five) repetitive domains of 110 amino acid residues, each containing a 30 amino acid subdomain with similarity to a sequence in the peptide-binding groove of the MHC class II β chain [43]. Secreted Eap can redock on the bacterial cell wall via cell wall-associated neutral phosphatase and this docking property enables Eap to promote adherence of *S. aureus* to host such as Fn and other extracellular matrix components [44] as well as cells, including fibroblasts and epithelial cells [45]. 

Emp (Extracellular matrix protein-binding protein) is a cell surface-associated protein of *S. aureus* with a broad spectrum of binding activity for extracellular matrix proteins such as fibrinogen, vitronectin, Fn, and collagen. The protein is expressed in the stationary growth phase suggesting that it could play roles in late infective processes. Emp binds the ligands with an affinity in the nanomolar range and the binding region has been tentatively located in the central part of the molecule [46,47].

Fn also functions as a linker between CD163, a multiligand scavenger receptor exclusively expressed by professional phagocytes, and *S. aureus* surface components. Soluble CD163 interacts with a subdomain of the gelatin/collagen binding region of Fn and, in turn, Fn binds to FnBPA or FnBPB. The formation of this three-component complex promotes phagocytosis and subsequent killing of bacteria by monocytes and granulocytes. Even non-professional phagocytes such as endothelial cells kill bacteria more efficiently if these are covered with soluble CD163. Thus, targeting pathogen-bound Fn with sCD163 would be a defense mechanism of the innate immune system against *S. aureus*. This finding is of particular importance in view of the fact that increased levels of soluble CD163 can occur during sepsis [48]. Therefore, in the presence of CD163, binding of Fn to FnBPA or FnBPB could result in an advantageous event for the host.

Fn-binding proteins with organization similar to FnBPA have been described in other staphylococcal species. *S. pseudintermedius* is an opportunistic pathogen that colonizes the nares, the perineum of healthy dogs, and the main cause of canine skin infections [49,50]. SpsD (*S. pseudintermedius* surface protein D) and SpsL (*S. pseudintermedius* surface protein L) are examples of MSCRAMMs that mediate the binding of *S. pseudintermedius* to fibrinogen and Fn. Domain binding to fibrinogen is located to the *N*-terminal region in both the proteins [51,52], while the Fn-binding is mediated by a segment that maps to the C-terminus of the proteins (Figure 2B,C) [53]. Both SpsD and SpsL play an important role in the internalization of *S. pseudintermedius* by host cells and, as reported for FnBPA, the process involves Fn and integrin α_5_β_1_ [53]. 

### 3.2. Fibronectin-Binding Proteins from Streptococci

#### 3.2.1. Fn-Binding Proteins from *Streptococcus pyogenes*

*Streptococcus pyogenes*, also known as group A *Streptococcus* (GAS), causes mild human infections such as impetigo and pharyngitis and serious infections such as necrotizing fasciitis and streptococcal toxic shock syndrome [54,55,56,57,58]. Moreover, repeated GAS infections may trigger autoimmune diseases, such as acute post-streptococcal glomerulonephritis, acute rheumatic fever, and rheumatic heart disease [59].

*S pyogenes,* expresses at least 12 distinct Fn-binding proteins [60] that can be divided in two subgroups. The first subset of adhesins contains cell wall anchored proteins including repetitive Fn-binding units. The most studied of these proteins is the Fn-binding protein F1 and its homologous Sfb1 protein (Streptococcus fibronectin binding protein 1) [60,61,62,63]. F1/Sfb1 contains a C-terminal series of between two and seven Fn-binding repeats homologous to those present in FnBPA or FnBPB of *S. aureus* that bind to the NTD of Fn (Figure 2D). As reported for FnBPA and FnBPB, this series of repeats is disordered and undergoes a disorder-to-order transition on binding the NTD according to the above described β zipper interaction [64]. F1 has also an upstream region (UR), immediately preceding the repetitive region, that shows the ability to bind the gelatin binding domain (GBD) of Fn (FI_6_ through FI_9_) [65,66]. The binding of F1 to Fn is proposed as a process in which the NTD initially forms a complex with the repetitive region of F1 followed by the cooperative activation and enhanced binding of UR to the GBD (Figure 1B). Unlike the repetitive region of FnBPA and FnFPB, which is more than sufficient for *S. aureus* adherence and internalization, the repeat region of F1 mediates adhesion of *S. pyogenes* to Fn in the extracellular matrix, while the UR of the protein triggers invasion of the host cells by this bacterium [67]. Interestingly, UR competitively inhibits incorporation of both cellular and plasma Fn into the extracellular matrix [68].

Fn-binding repeats with homologous sequences to those present in F1 and FnBPA and FnBPB have been found in other streptococcal Fn-binding proteins such as F2, FbaA and FbaB, SOF (Serum Opacity Factor) and SfbX (*Streptococcus* fibronectin-binding protein X). F2 is similar to Fn-binding proteins FnBB from *Streptococcus dysgalactiae* and FnB from *Streptococcus equisimilis* and unlike protein F1 lacks the UR for binding the GBD of Fn [69]. FbaA, a 38 kDa protein, contains three/four proline rich-repeat motifs and shows considerable homology to FnBPA of *S. aureus*, while its possession of Fn-binding repeats is unclear [70]. FbaB is significantly homologous to the C-terminal portion of F2 and contains Fn-binding repeats [71]. Both FbaA and FbaB also promote Fn-dependent internalization of streptococci in host cells [71,72].

SOF is a bifunctional LPXTG adhesin which contains two major domains, a large *N*-terminal domain that mediates opacification of sera and a C terminal repetitive region that targets the *N*-terminal domain of Fn [73].

The *sfbX* genes from different GAS serotypes occur immediately downstream of the *sof* gene and encode 650 residue surface-bound proteins sharing about 90% sequence identity. SfbX residues, approximately one to 480, are not particularly similar to those of other known proteins, with the closest match being the *S. aureus* coagulase protein. The remaining portion of these proteins (residues 481–650) contains four Fn-binding repeats highly similar to those of other streptococcal Fn-binding proteins and a potential LPXSG cell wall anchor motif [74]. 

A second subgroup of multifunctional adhesins of *S. pyogenes* possesses the ability to bind Fn but lacks Fn-binding repeat units and includes some M proteins (M1, M3, and M6), GAPDH, protein H, Shr, Scl1, and Fbp54.

Direct binding of Fn to M proteins allows Fn to interact with the host cell surface integrin α_5_β_1_. This bridging activity of Fn between M protein on bacterial cells and α_5_β_1_ integrin on the surface of host cells promotes internalization of bacteria into the host cells [75,76].

Protein H, known for its ability to interact with immunoglobulins, also binds to the FnIII of the N-CAM and the central cell-binding domain of Fn. Since protein H/Fn interaction is not disrupted by RGD peptide, it is plausible that protein H does not bind to FnIII_10_ [77].

Shr (Streptococcal hemoprotein receptor) is a 145 kDa protein firmly attached to the bacterial surface. Shr binds haemoglobin and haemoglobin-haptoglobin complex [78] and also interacts with Fn and laminin, suggesting that it also acts as an adhesin [79].

Two other major adhesins are the streptococcal collagen-like proteins 1 (Scl1) and 2 (Scl2). Scl1 is an LPXTG homotrimeric protein with an *N*-terminal globular domain, followed by a collagen-like domain and a C-terminal cell wall-anchoring domain [80] (Figure 2E). Unlike many other bacterial adhesins, Scl1 specifically binds to the alternatively spliced extra modules A (EDA) and B (EDB) of cellular Fn through conserved structural determinants present within Scl1 globular domain (Figure 1B) [81]. Importantly, interaction of Scl1 with EDA expressed in wounded tissue environments facilitates biofilm formation on extracellular matrices and implements GAS pathogenesis [82,83].

GAPDH (glyceraldehyde-3-phospate dehydrogenase) is tightly attached to the surface of streptococci and binds to Fn, lysozyme, and the cytoskeletal proteins myosin and actin [84].

Fbp54 (Fibronectin-binding protein 54), a 54 kDa protein, interacts with the NTD of Fn and the Fn-binding domain of Fbp54 has been localized to the first 89 *N*-terminal residues of the protein [85]. Fbp54 is expressed in vivo, is immunogenic in the human host, and mediates adhesion of *S. pyogenes* to buccal epithelial cells [86].

#### 3.2.2. Fn-Binding Proteins from Other Streptococcal Species

*Streptococcus pneumoniae* is a human-specific pathogen and recognized as the main cause of community acquired pneumonia and meningitis in children and the elderly, and of sepsis in children worldwide [87,88]. *S. pneumoniae* produces several Fn-binding proteins. PavA (Pneumococcal adherence and virulence factor A), is homologous to Fbp54 of *S. pyogenes* (67% identity) and to FbpA of *S. gordonii* (74% identity) and consists of 551 amino acid residues [89]. PavA is a cell surface non-LPXTG protein binding to surface-coated Fn, but not to soluble Fn [90].

A second pneumococcal Fn-binding protein is the LPXTG protein PavB. In its mature part, PavB consists of typical repetitive domains referred to as Streptococcal SUrface REpeats (SSURE) (152 amino acidic residues) and each highly conserved core SSURE bears no significant sequence similarity to proteins of known function [91,92]. PavB has the ability to interact directly via a still unknown host cell receptor or with extracellular matrix proteins such as Fn, plasminogen or thrombospondin-1 [91,92,93,94]. PavA and PavB binding is specifically mediated by defined interactions with FnIII repeats [95].

An additional LPXTG surface protein, termed PfbA (Plasmin and fibronectin-binding protein A), binds with moderate affinity several plasma proteins including Fn, plasminogen/plasmin, and albumin [96]. The crystal structure of the large recombinant fragment, PfbA_150–607_, revealed it to possess a beta-helical region followed by a C-terminal disordered segment with structural and sequence features that resemble the Fn-binding regions of FnBPA of *S. aureus* and BBK32 of *Borrelia burgdorferi* [97]. Recently, it has been reported that the open reading frame spr0075 encodes for a 120 kDa protein, named plasminogen/fibronectin-binding protein B (PfbB), which displays an LPXTG cell wall anchoring motif and six repetitive domains that directly interact with Fn, allowing adherence of *S. pneumoniae* to various epithelial respiratory tract cell lines [98]. 

RrgA is an 893-residue elongated macromolecule localized at the pilus 1 tip. Its fold contains four nested domains (D1 to D4) presenting both eukaryotic and prokaryotic origins [99] and playing a direct role in binding to epithelial cells and to laminin and fibronectin [100,101]. It has been proposed that full length RrgA binds to Fn either through single D3 or D4 domains or both domains simultaneously in a dual domain binding mode [102].

*Streptococcus gordonii* is a member of the viridans group of oral bacteria and considered an important agent of dental caries, gingivitis, and periodontitis [103]. Similar to many streptococci, *S. gordonii* expresses a protein named FbpA (Fibronectin-binding protein A) that has high sequence identity to PavA of *S. pneumoniae* and Fbp54 of *S. pyogenes* and interacts with Fn [104]. *S. gordonii* expresses other proteins such as surface SspI/II (also named SspA/B) [105] and CshA and CshB [106,107], all exhibiting Fn-binding capacity. CshA binds to Fn via an *N*-terminal non repetitive region, a part of the protein composed of three distinct segments named NR1, NR2, and (Figure 2F) [108]. According to a model proposed by Back et al. NR1 acts to ”catch” Fn forming a readily dissociable pre-complex, which is subsequently stabilized by a high affinity binding mediated by the contiguous NR2 (“catch-clamp” mechanism) [108].

*Streptococcus mutans* is a pioneer oral bacterium that is recognized as an agent of bacterial endocarditis. *S. mutans* possesses both SspI/II and a homologue of PavA, named SmFnB. Inactivation of the gene for SmFnB impairs the adherence of *S. mutans* to Fn [109].

*Streptococcus suis*, a microorganism causing infectious diseases in pigs and other domestic animals [110], expresses a PavA homologue named FbpS (Fibronectin-binding protein of *S. suis*) (70% identity with PavA) that interacts with fibrinogen and Fn [111] and the Fn-binding site has been located in the N terminal region of the protein [112]. Other Fn-binding proteins expressed by *S. suis* are enolase [113] and OFS (Opacity Factor from *S. suis*) [114].

*Streptococcus agalactiae* (Group B Streptococcus (GB)) colonizes the human lower intestinal and genital tracts and constitutes a major threat to neonates from pregnant carrier mothers and to adults with underlying morbidity. ScpB (streptococcal C5a peptidase B) is a 120 kDa multidomain protein composed of an *N*-terminal protease domain followed by a domain containing three Fn type III modules (Fn_1–_Fn_3_) at the C-terminus. Notably, the protease domain is interrupted by an insertion named the protease-associated (PA) domain of unknown function (Figure 2G) [115]. ScpB also binds with high affinity to surface-coated but not soluble Fn [116]. It has been proposed that this preferential binding could be due to the formation of determinants produced by the flanking of several Fn molecule on a solid surface [117]. The finding that a natural variant of ScpB containing a 4-amino-acid deletion that eliminates peptidase activity still maintains the capability to bind Fn, indicates that the Fn-binding activity resides in a different region from the protease domain [118].

### 3.3. Fn-Binding Proteins from Other Gram-Positive Bacteria

#### 3.3.1. Enterococci

*Enterococcus faecium* has emerged over the past three decades as an agent capable of causing life-threatening infections including infective endocarditis [119].

As reported for *S. pyogenes* and *S. pneumoniae*, *E. faecium* expresses a considerable number of Fn-binding adhesins. SagA (Secreted antigen A) is an 80–90 kDa protein exhibiting the capability to bind extracellular matrix proteins such as fibrinogen, collagens, laminin, and Fn. The SagA is organized into three domains: i) A putative coiled-coil *N*-terminal domain; ii) a central domain containing direct repeats; iii) a C-terminal domain [120]. A second adhesin identified in *E. faecium* is Fnm (Fibronectin-binding protein of *Enterococcus faeciu*m), a homologue of *S. pneumoniae* PavA, that promiscuously binds to fibronectin, collagen type V, and laminin [121]. Binding to Fn is mediated by the *N*-terminal region of Fnm. *E. faecium* also expresses a group of six proteins containing the WxL motif (a short sequence that confers a cell surface localization function), which share the ability to bind type I collagen and Fn [122]. Lastly, this bacterial species expresses a peptiglycan-anchored protein named PrpA (Proline-rich protein A) which contains a unique *N*-terminal domain and a C-terminal proline-rich region homologous to the *Streptococcus agalactiae* surface protein BibA. The recombinant *N*-terminal domain of PrpA shows the ability to interact with Fn and fibrinogen [123].

*Enterococcus faecalis* contributes to a number of infections in humans including bacteremia, abdominal and pelvic infections, urinary tract infections, and septicemia [124]. *salA* and *salB* are two *sagA*-like genes which encode proteins that are antigenic during infection and bind to collagen type I and Fn [125]. *E. faecalis* also expresses the anchorless PavA-like adhesion EfbA (Enterococcus fibronectin-binding protein A), a protein that binds with high affinity to immobilized Fn, collagen I, and collagen V [126,127].

#### 3.3.2. Mycobacteria

This genus includes pathogens known to cause serious diseases in mammals, including tuberculosis (*Mycobacterium tuberculosis*) and leprosy (*Mycobacterium leprae*) in humans. Mycobacteria have a variety of Fn-binding proteins the most important of which is the antigen 85 complex. The complex consists of three proteins named 85A, 85B, and 85C, encoded by three genes located in different sites in the mycobacterial genome and showing homology and extensive immune cross-reactivity. The proteins differ slightly in molecular mass ranging from 30 to 31 kDa. Depending on the mycobacterium species, the binding site on Fn varies from the gelatin-binding domain [128] to the *N*-terminal binding domain [129]. In the antigen 85B two short motifs corresponding to the sequences 84–110 and 211–230 were identified and shown to have Fn-binding activity. Moreover, inside the 85–110 aa residues a unique six amino acid sequence (FEWYYQ) was shown to be crucial for Fn-binding. This segment forms a helical structure and is exposed at the surface of the protein. Another mycobacterial Fn-binding protein is MPB51 encoded adjacent to the gene 85A and showing 40% identity to other members of the antigen 85 complex [130]. A third Fn-binding protein identified in several species of mycobacteria (among other, *M. tuberculosis*, *M. leprae*, and *M. bovis*) is FAP (Fibronectin Attachment Protein) [131,132]. The binding site for FAP was localized in the NTD of Fn, while the FAP sequence essential for Fn-binding was mapped to the sequence 269–280 (GNRQRWFVVWLG) [133]. 

An additional Fn-binding protein is malate synthase, a cytoplasmic enzyme involved in the glyoxylate pathway and when associated to the cell wall it is capable of interacting with Fn and laminin. The binding site for Fn is located in the C-terminal end of the enzyme, while no information is available on the malate synthase binding site in Fn [134].

#### 3.3.3. *Listeria Monocytogenes*

*Listeria monocytogenes* is the agent of listeriosis, a disease that causes brain abscess, meningitis and septicemia mainly among pregnant women, unborn babies, newborns, and immunocompromised patients. Five fibronectin-binding proteins with a mol. weight ranging from 55 to 27 kDa were identified in *L. monocytogenes*. The 55 kDa protein was proved to be present on the bacterial cell surface [135]. A gene encoding a 24.6-kDa Fn-binding protein (Fbp) was also isolated and sequenced. The *fbp* gene was found to be present in all tested isolates of the species *L. monocytogenes* and no homologies between the *fbp* gene and its translation product with any other DNA or proteins deposited in databanks were found [136].

A gene named *fbpA*, required for efficient liver and intestine colonization of mice was also identified. *fbpA* was found to encode a 570-amino-acid polypeptide that has strong homologies to typical fibronectin-binding proteins. FbpA is bound to immobilized human fibronectin in a dose-dependent and saturable manner and increases adherence of wild-type *L. monocytogenes* to HEp-2 cells in the presence of exogenous fibronectin [137].

#### 3.3.4. Clostridia

*C. difficile*, an anaerobic toxigenic bacterium, causes severe infectious colitis that leads to significant morbidity and mortality worldwide. A gene termed *fbp68* encoding a 68 kDa protein with an Fn-binding activity has been identified in *C. difficile*. The Fbp68 protein (Fibronectin-binding protein 68) is 68% identical to *S. pyogenes* Fbp54 and found on the cell surface, and is shown to bind to both soluble and insoluble Fn [138]. The pathogen *C. perfrigens* is a common cause of wound-associated infections and food poisoning. Genomic analysis of *C. perfrigens* revealed that this bacterium contains two genes that encode putative Fn-binding proteins FbpA and FbpB, having molecular masses of 25 and 66 kDa, respectively. The FbpB is homologous to the 68 kDa protein from *C. difficile* and recognizes the FnIII_9_–FnIII_10_ region of Fn [139,140].

In Table 1 molecular properties of the mostly known adhesins from Gram-positive bacteria are reported.

### 3.4. Fn-Binding Proteins from Other Gram Negative-Bacteria

#### 3.4.1. *Escherichia coli*

A very heterogeneous class of Fn-binding proteins has been described in *E. coli* species. Two types of Fn receptors have been described in the enterotoxigenic strains of *E. coli* [141], while uropathogenic *E. coli* (UPEC) utilize two type I fimbriae to bind Fn [142]. Furthermore, UPEC express a large surface protein that binds to both laminin and Fn and belongs to the family of trimeric autotransporters [143].

Certain strains of Enterobacteriaceae express amyloid fibers named curli that promote Fn-mediated internalization and this process is blocked by RGD peptide, suggesting that curli mediate binding to FnIII_10_ of Fn [144], while the pili of enterohaemorrhagic (EHEC) *E. coli* called haemorrhagic coli pili (HCP) mediate binding to laminin and Fn [145]. 

#### 3.4.2. *Campilobacter Jejuni*

*C. jejuni* is the causative agent of enteritis, characterized by abdominal pain, fever and diarrhea. The main *C. jejuni* Fn adhesin is CadF (*Campilobacter* adhesin to Fn). CadF is 300 aa protein with a domain organization and sequence identity (30%) to members of the OmpA/prF porin family suggesting that it may also function as a porin.

The CadF Fn-binding site resides in a protein region including the FRLS (phenylalanine-arginine-leucine-serine) motif in position 134–137 [146]. In accordance with this, peptides containing the FRLS bind to Fn and inhibit binding of CadF to Fn and epithelial cell monolayers. A second Fn-binding protein identified in *C. jejuni* is FlpA (Fibronectin-like protein A), a protein which contains three FIII modules [147] and involved in bacterial attachment to epithelial cells [148]. CadF and FlpA cooperate along with several secreted proteins for optimal promotion of *C. jejuni* invasion of host cells [149].

#### 3.4.3. *Salmonella enterica* Serotype Typhimurium

*Salmonella* species cause a wide range of disease in multiple hosts. *Salmonella enterica* serovar Typhimurium causes self-limited intestinal disease in humans and systemic typhoid-like illness in susceptible mice. Two Salmonella monomeric autotransporter proteins have been shown to function as Fn-binding proteins: ShdA and MisL. Kingsley et al. demonstrated that ShdA is a large outer membrane protein that specifically recognizes and binds to Fn and revealed that it was the passenger region of the protein (aa 59–1553) that interacts with this ligand [150]. The passenger domain contains a non-repeated region (aa 59–470) and a repetitive domain (470–1553). The repetitive region consists of two types of imperfect repeats, named repeat type A and B, respectively. The type A repeat is around 100 aa in length and is repeated three times, while the B repetitive unit (about 60 aa residues) is repeated nine times (Figure 2H). It has been shown that repeats A2, B8, A3, and B9 contribute to the formation of the F-binding site in ShdA, while the binding site for the active ShdA has been localized in the FnIII_13_ module of Fn, which is also part of the heparin-2-binding domain of Fn. Due to the presence of six positive charged aa residues that form the “cationic cradle” in FnIII13 and on the basis of mutagenesis studies it has been hypothesized that ShdA and Fn reciprocally contact through the salt-bridge dependent interaction [151,152].

MisL is the second, ShdA-related, autotransporter Fn-binding protein expressed by *S. enterica*. As reported for ShdA the Fn-binding site in MisL has been localized in the passenger region of the bacterial protein; however, the absence of the repeats involved in ShdA binding to Fn, indicates that the Fn-binding site in MisL is structurally different [153].

#### 3.4.4. *Borrelia burgdorferi*

*Borrelia burgdorferi*, is a pathogenic spirochete endemic to North America and the causative agent of Lyme disease. Lyme disease is characterized by severe clinical complications such as arthritis, carditis, and neurological dysfunction and often is accompanied by a bull eye-shaped rash (erythema migrans lesion) surrounding the area of infection [154,155].

BBK32, a 47 kDa Fn-binding lipoprotein from *B. burgdorferi*, contributes to colonization, dissemination, and infection [156,157]. BBK32 has a non-repetitive Fn-binding sequence and is linked to the bacterial cell surface by the *N*-terminal end. The N-terminus of BBK32 contains a signal peptide followed by a ‘‘lipobox’’ and an extended, non-repetitive intrinsically disordered segment (aa residues 21–205) which contains the Fn-binding sites (Figure 2I) [158]. This segment contains a motif (aa 147–205) that binds to the *N*-terminal type I modules found in Fn by the tandem-β zipper mechanism (Figure 1B) [29], BBK32 also includes a putative gelatin-binding sequence corresponding to residues 120–147 that resembles the GBD-binding sequence found in protein F1 (Figure 1B) [159]. 

Like anastellin, BBK32 can induce conformational changes in soluble Fn that can lead to the ordered aggregation of the glycoprotein (superfibronectin) which exposes thermolysin cleavage sites that are cryptic in soluble Fn. The superfibronectin-forming activity, localized to a sub-region between residues 160 and 175 of BBK32, targets FIII_1–3_ modules in the central domain of Fn (Figure 1B).

Lastly, recombinant BBK32 can affect the structure of Fn matrices formed by cultured fibroblasts and effectively inhibits endothelial cell proliferation [159].

*B. burgdorferi* can invade and retain viability in nonphagocytic cells in a process that requires β1 integrins and Src kinase activity but not BBK32.The negative involvement of BBK32 could be due to the fact that, while adhesins such as FnBPA contain multiple binding sites on the NTD of Fn and promote integrin clustering and subsequent invasion by these pathogens, BBK32 contains a sole NTD binding site and, as such, lacks the ability to cluster enough integrins to trigger the signaling pathway needed for invasion of the host cells [160].

*B. burgdorferi* codes for other known Fn-binding proteins such as BB0347 [161] and RevA [162], but the molecular details of their interactions with Fn have still to be studied in detail.

Table 2 reports molecular properties for the mainly known adhesins from Gram-negative bacteria.

## 4. Fn-Binding Proteins as Virulence Factors

Virulence factors refer to gene products that facilitate the fitness of a bacterium and enable a microorganism to enhance colonization and infection in the host. Virulence factors include toxins, surface/secreted proteins that mediate bacterial attachment or that protect a bacterium from innate and adaptive immunity and hydrolytic enzymes that may contribute to the pathogenicity of the bacterium. For many infectious diseases, the mouse has emerged as an ideal animal model due to low cost, small size, ease of handling, and ability to reflect several aspects of disease progression in humans. Moreover, experimental murine models have been constructed to test the potential virulence of a bacterial factor in different infectious contexts such as bacteremia, sepsis, peritonitis, and endocarditis. As reported above, Fn is largely abundant in animal tissues and fluids and potentially serves as a substrate for colonization/invasion. Furthermore, exposure of microbes to biological fluids may allow them to evade recognition by a host defense mechanism. Consequently, Fn-binding proteins on bacterial surfaces may serve both as attachment and anti-opsonic factors and have good potential as virulence determinants. To experimentally prove this concept, isogenic mutants defective in the adhesin of interest have been generated and virulence of the strain has been compared to the wild-type in a suitable animal infection model. This genetic approach provided important information on the role of a specific Fn-binding protein as a virulence factor. However, as with all experimental systems, it has some limitations: In fact, functional redundancy sometimes makes it difficult to show conclusively that a mutant lacking a single factor has reduced virulence. For this reason, other experimental approaches, such as the expression of an Fn-binding protein in a surrogate host like *Lactococcus lactis* or *Staphylococcus carnosus*, have been proposed. Several cases can be taken from scientific literature, in which Fn-binding proteins were assessed as virulence factors. For example, transposon mutant of *S. aureus* with reduced Fn-binding capacity showed reduced adherence to traumatized rat heart valve [163]. Along this line, the heterologous expression of FnBPA in *L. lactis* significantly decreased the *inocula* number required to cause endocarditis. In a later work, it turned out that the valve infectivity was due to the fibrinogen-binding domain of FnBPA, but not to the repetitive Fn-binding region [164]. More conclusively, it was found that lactococci expressing both FnBPA and FnBPB produce a 50–100 increase in infection compared with untransformed lactococci [165] and that fibrinogen-and Fn-binding domains of FnBPA synergistically promote endothelial invasion and endocarditis [166,167]. Likewise, inactivation of the gene encoding FnbA from *S. pyogenes* resulted in a significantly reduced cell adherence to and invasiveness of Hep-2 cells and demonstrated decreased mortality compared with the wild-type FbaA-bearing GAS in the murine skin infection model [70]. Similar attenuation of virulence was observed with mice challenged intraperitoneally with *sof*-defective strains of GAS [168] and when zebrafish was intramuscularly injected with *shr* mutant of GAS [79]. A transposon mutant of *S. sanguis* with decreased adherence to surface-coated Fn, also showed reduced virulence in a rat model of infectious endocarditis [169]. Moreover, the *bbk32* knockout mutant of *B. burgdorferi* exhibited reduced Fn-binding in solid phase assays and manifested decreased interactions with fibroblasts. In accordance with this, virulence of the mutant was significantly attenuated in the murine model of Lyme disease relative to genetically complemented control [170]. Lastly, in a mouse model of *S. typhimurium* intestinal persistence, a *misL* mutant was shed with the feces in significantly lower numbers than the wild type and was impaired in its ability to colonize the cecum [153].

In regards to implant-associated infection, pioneering experimental works dealt with the adhesion of staphylococci to implant surfaces and the inhibition of bacterial adhesion by heparin surface-modification [171]. Then, the binding of staphylococci to fibronectin, which covers implants in vivo, and the effect of heparin were studied by dynamic force spectroscopy, demonstrating a specific inhibition by heparin of the *Staphylococcus*-fibronectin interaction at the molecular binding site of fibronectin [172]. These findings explained and substantiated the previous observations on the ability of heparin surface-modification of biomaterials to prevent bacterial adhesion. Furthermore, the finding that, among the numerous adhesins studied, those of fibronectin were detectable in most clinical isolates of *S. aureus* from implant-related infections, highlighted the weight of fibronectin-binding adhesins as virulence factors in clinical implant infections [173,174].

Identification of a Fn-binding protein as a virulence factor can be also attained by immunological approaches in which protection from bacterial infection is examined, targeting a specific adhesin by vaccination. For example, immunization of mice with Sfb1 adhesin resulted in a protective response against *S. pyogenes* after intranasal vaccination of mice [175]. Likewise, mice immunized subcutaneously or orally with Fbp54 protein survived significantly longer following the challenge with *S. pyogenes* than non-immunized mice, suggesting that Fbp54 may be a promising vaccine candidate [176]. In conclusion, these data support the notion that the expression of Fn-binding proteins is an important part of the virulence of bacteria and that reduced adhesion is associated with a reduction in virulence.

## 5. Targeting Fn-Binding Proteins as a Means of Infection Control

Targeting bacterial colonization could be therapeutically useful in the light of prevailing antibiotic resistance and this goal can be achieved by vaccination (active immunization) or administration of Fn adhesin-specific antibodies (passive immunization). An effective adhesin-based vaccine contains a specific bacterial surface protein, or a recombinant part of it, which, when injected, induces a specific immune response and protection of subjects against later exposure to a pathogen. Conversely, in passive immunization, preformed exogenous antibodies against a specific antigen are transferred from an immune individual to a non-immune recipient by natural or artificial transfer.

Adhesin-based antibodies participate in host defense in several ways: a) By binding and blocking the action of an adhesin; b) by direct opsonization of bacteria, facilitating recognition, and ingestion of bacteria by neutrophils and macrophages; c) by forming a complex with bacterial surface adhesins, with said complex triggering the activation of the complement system and consequent killing by professional phagocytes.

Although the use of Fn–binding proteins as a vaccine component is not necessarily the best option [31,177], in a number of cases, vaccination with purified Fn-binding proteins has provided efficient protection against bacterial infection in animal models. For example, the growth of wild-type bacteria in whole human blood containing anti-PfbA antibodies was significantly reduced compared with its growth without the antibody, suggesting that PfbA is an important factor in the development of pneumococcal infections and that it could be a potential candidate vaccine against *S. pneumoniae* [96].

Vaccination of rats with purified recombinant EfbA protein from *E. faecalis* provided protection against endocarditis and demonstrated that EfbA immunization is effective in preventing this infection, possibly by interfering with bacterial adherence [127].

Data reported by Brown et al. demonstrate that BBK32 injected along with other surface proteins such as DbpA and OspC dramatically impacts vaccine efficacy when tested in experiments to provide protection against *Borrelia* [178].

Furthermore, immunization with genetically modified and attenuated *Salmonella enterica* strain harboring *shdA* and *misL* afforded complete protection against challenge with a virulent strain of serotype Typhimurium [179].

Together, these data demonstrate that Fn-binding proteins have potential as a vaccine component against both Gram-positive and Gram-negative bacteria.

## 6. Concluding Remarks and Outlook

A detailed biochemical analysis and the systematic use of structural biology has increased the appreciation of Fn interaction with bacterial adhesins and has facilitated the development of mechanistic models (for example, the β zipper model) of Fn binding to specific bacterial receptors. Additionally, several Fn-binding proteins have been investigated as potential antigens for vaccination to protect against infection and have been shown to offer protection in murine models. In view of these convincing successes, many aspects of biochemistry, immunology and pathology of a large number of bacterial Fn-binding adhesins remain unclear and incomplete. For example, most work on Fn-binding proteins has been confined to laboratory strains. Further analysis is required with regard to clinical isolates, which may present considerable variations both in the repertoire of Fn-binding proteins as well as in the sequence of binding domains. Furthermore, in many cases, the timing of expression in vivo during the growth phases as well as the genetic mechanism for regulating Fn-binding proteins are still unknown.

As noted above, the administration of a bacterial Fn receptor as a vaccine may not be effective enough to protect animals. Recombinant fragments of bacterial adhesins should, therefore, be genetically manipulated to improve their antigenic potential.

We should also bear in mind that several bacterial species redundantly express a multitude of Fn-binding proteins. Therefore, in order to formulate the composition of an effective vaccine against a pathogen it is essential to select an Fn adhesin that is robustly expressed in vivo and shows high antigenic potential and an effective role in the pathogenesis of the infection.

As the development of an infection is often a multifactorial process, an Fn-binding protein tested as a vaccine component in preclinical trials generates only partial protection in animal infection models. Therefore, when a new multivalent vaccine is designed, the selection of appropriate antigens (Fn-binding adhesins, toxins, immune evasion factors, etc.) must be carefully assessed.

Lastly, caution should be taken when results obtained with animal models are automatically extended to infectious human diseases caused by the same pathogen. Preclinical animal models do not necessarily reflect the infection process observed in humans due to the different biochemical and immunological context encountered by the infecting microorganism.

With this in mind, Fn-binding proteins offer a great chance of success in the development of therapies against several human pathogens.

## Figures and Tables

**Table 1:** Molecular properties of Gram-positive Fn-binding proteins.

Adhesin	Host	Mass (kDa)	Fn Site	Binding Mechanism	Refs
FnBPA-FnBPB	*Staphylococcus aureus*	106–104	*N*-terminal domain	β-zipper	[28,31]
Ebh	*S. aureus*	1100	?	?	[42]
Eap (MAP)	*S. aureus*	15	?	?	[44]
Emp	*S. aureus*	36	?	?	[46,47]
SpsD	*Staphylococcus pseudintermedius*	114	*N*-terminal domain	?	[53]
SpsL	*S. pseudintermedius*	102	*N*-terminal domain	?	[53]
F1/Sfb1	*Streptococcus pyogenes*	69	*N*-terminal domain Gelatin-binding domain	β-zipper	[64,65,66]
F2	*S. pyogenes*	128	*N*-terminal domain	?	[69]
FbaA	*S. pyogenes*	38	?	?	[70]
FbaB	*S. pyogenes*	81	?	?	[71]
SOF	*S. pyogenes*	112	*N*-terminal domain	?	[73]
SfbX	*S. pyogenes*	82	?	?	[74]
Protein H	*S. pyogenes*	37	Central-binding domain	?	[77]
Shr	*S. pyogenes*	143	?	?	[79]
Scl1	*S. pyogenes*	46	EDA-EDB	?	[80,81]
Fbp54	*S. pyogenes*	54	*N*-terminal domain	?	[85]
PavA	*Streptococcus pneumoniae*	63	Central-binding domain	?	[89,95]
PavB	*S. pneumoniae*	107	Central-binding domain	?	[95]
PfbA	*S. pneumoniae*	74	?	?	[96,97]
PfbB	*S. pneumoniae*	120	?	?	[98]
RrgA	*S. pneumoniae*	98	?	?	[100,101]
FbpA	*Streptococcus gordonii*	63	?	?	[104]
CshA	*S. gordonii*	265	?	catch-clamp	[108]
FbpS	*Streptococcus suis*	64	*N*-terminal domain	?	[111,112]
ScpB	*Streptococcus agalactiae*	126	?	?	[116,117]
SagA	*Enterococcus faecium*	52	?	?	[120]
Antigen 85A Antigen 85B Antigen 85C	*Mycobacteria* spp.	31 30 31	*N*-terminal domain Gelatin-binding domain	?	[128,129]
FAP	*Mycobacteria* spp.	30	*N*-terminal domain	?	[131,132,133]
FbpB	*Clostridium perfrigens*	66	FnIII_9_-FnIII_10_	?	[139,140]

**Table 2:** Molecular properties of Gram-negative Fn-binding proteins.

Adhesin	Host	Mass (kDa)	Fn Site	Binding Mechanism	Refs
Curli	*Escherichia coli*	?	FnIII_10_	?	[144]
CadF	*Campilobacter jejuni*	34	?	?	[146]
FlpA	*C. jejuni*	46	?	?	[147]
ShdA	*Salmonella enterica* serovar Typhimurium	207	FnIII_13_	?	[150,151,152]
MisL	*S. enterica* serovar Typhimurium	101	?	?	[153]
BBK32	*Borrelia burgdorferi*	47	*N*-terminal domain Gelatin-binding domain FIII_1–3_	β-zipper	[158,159]
